# The Relevance of Variants With Unknown Significance for Autism Spectrum Disorder Considering the Genotype–Phenotype Interrelationship

**DOI:** 10.3389/fpsyt.2019.00409

**Published:** 2019-06-07

**Authors:** Diogo V. Lovato, Roberto R. Herai, Graciela C. Pignatari, Patricia C.B. Beltrão-Braga

**Affiliations:** ^1^Laboratory of Disease Modeling, Department of Microbiology, Institute of Biomedical Sciences, University of São Paulo, São Paulo, Brazil; ^2^Experimental Multiuser Laboratory, Graduate Program in Health Sciences, School of Medicine, Pontifícia Universidade Católica do Paraná, Curitiba, Brazil; ^3^Lico Kaesemodel Institute (ILK), Curitiba, Brazil; ^4^Laboratory of Disease Modeling, Scientific Platform Pasteur-USP, São Paulo, Brazil

**Keywords:** autism spectrum disorders, variants of unknown significance, next-generation sequencing, de novo mutation, rare variants

## Abstract

Several efforts in basic and clinical research have been contributing to unveiling the genetics behind autism spectrum disorders (ASD). However, despite these advancements, many individuals diagnosed with ASD and related neuropsychiatric conditions have been genetically investigated without elucidative results. The enormous genetic complexity of ASD-related conditions makes it a significant challenge to achieve, with a growing number of genes (close to a thousand) involved, belonging to different molecular pathways and presenting distinct genetic variations. Next-generation sequencing (NGS) is the approach most used in genetic research related to ASD, identifying de novo mutation, which is closely related to more severe clinical phenotypes, especially when they affect constrained and loss-of-function intolerant genes. On the other hand, de novo mutation findings contribute to a small percentage of the ASD population, since most of the cases and genetic variants associated with neuropsychiatric conditions are inherited and phenotypes are results of additive polygenic models, which makes statistical efforts more difficult. As a result, NGS investigation can sound vainly or unsuccessful, and new mutations on genes already related with ASD are classified as variants of unknown significance (VUS), hampering their endorsement to a clinical phenotype. This review is focused on currently available strategies to clarify the impact of VUS and to describe the efforts to identify more pieces of evidence throughout clinical interpretation and genetic curation process.

## Introduction

Autism spectrum disorder (ASD) is a complex neurodevelopmental disorder (ND), usually identified during early childhood according to clinical criteria, comprising impairment of the social abilities and cognitive competence, as well as typical behavior including stereotypic movements. Despite the criteria used to categorize someone under ASD’s umbrella, it is well known that ASD is not homogeneous in terms of clinical signs and genetic aspect, being close to having one thousand genes already implicated (1, 2). Considering that, it is quite evident that not all of the ASD subtypes had the same origin, and depending on the genetic background of the person and environmental factors involved (3, 4), clinical phenotype could be extremely different and could vary significantly, especially in terms of severity (5, 6).

Based on the challenging efforts to better characterize the ASD genetic background, several studies concentrate their energies to investigate and correlate the genetic profile of different ASD individuals. These investigations allowed the determination that only a small proportion of ASD cases have a clear-signed mendelian genetic background, while most of the current cases are caused by a complex combination of polygenic additive effect, comprising an enigma in terms of how these genetic variations are combined to manifest the autistic phenotypes (7, 8). Due to the genotypic diversity and enormous possibility of genetic variations among individuals within the spectrum, it is challenging to identify and correlate specific genetic variation for each and its relationship with ASD etiology, not only considering statistical issues but also how genetic mutations interfere in protein function and molecular pathways during early developmental stages (9).

Current investigations, both in clinical routines and research, also involve the use of next-generation sequencing (NGS) technologies to extract and sequence the entire genome (whole genome sequencing—WGS) or exome (whole exome sequencing—WES) of ASD individuals. These approaches usually imply in the identification of several genetic modifications described as variants of unknown significance (VUS), which effectively represent mutations where the pathogenicity and the function of the gene involved is unclear, turning it into a critical issue since most patients remain without a genetic explanation for their condition (10, 11). Thus, these VUS are genetic variants which are genes are already associated with ASD, but the specific altered regions do not have sufficient clinical evidence or functional shreds of evidence in order to be categorized in a pathogenic or benign variant. In other situations, some VUS seems to be relevant for clinical phenotype, but they are located at genes with few associations with ASD (12, 13). Even though in some cases researches could probably correlate some VUS and suggest pathogenicity, this is barely possible, claiming for a robust collection of evidence and data from the literature to support it, coming from research groups and laboratories around the world. This review presents the most recent knowledge regarding different strategies to obtain information from current VUS, during NGS curation and interpretation process, from ASD and related neuropsychiatric cases.

## Genetic Behind ASD

Most ASD and associated developmental delay cases are a genetical combination of inherited common and rare genetic variants. On the other hand, non-inherited genetic mutations are the most clinically relevant alterations with higher morbidity when affecting constrained genes associated with neurodevelopmental biology (14–18).

During NGS analysis and interpretation, it is essential to consider all genes and molecular pathways involved in the early stages of neurodevelopment. As demonstrated in several groups, genes expressed during neurogenesis and involved pathways are the most critical genes affected in ASD etiology. Particular examples of genes that are well established to be associated with synaptic function include GRIN2B (glutamate receptor ionotropic NMDA type 2B) and SHANK3 (SH3 And Multiple Ankyrin Repeat Domains 3), and associated with chromatin regulation include CHD2 (Chromodomain Helicase DNA Binding Protein 2) and CHD8 (Chromodomain Helicase DNA Binding Protein 8) (19–21). These genes have been significantly studied and have had hundreds of genetic variants described. Even though, like in other ASD associated genes, the relevance of each VUS should be investigated and enlightened considering patient’s phenotype, which would be very useful to proceed with research, clinical assessments, and genetic counseling. Additionally, the systematic identification of VUS could contribute to elucidate other NDs, including intellectual disabilities (IDs) and attention-deficit hyperactivity disorder (ADHD), schizophrenia, mood disorders and others, as well as ASD comorbidities, which could be related to genes and pathways that are strictly connected as a broader cognitive spectrum (22, 23). As many ASD associated genes are essential not only for nervous system development but also for other biological processes, many health conditions could escort ASD cases. Current genetic knowledge demonstrates the relevant overlap between several genes associated with ASD and some diseases (17, 18). For example, several genetic variants in voltage-gated ion channels associated with ASD also cause diseases, such as epileptic encephalopathies, different kinds of ataxias, heart diseases, and psychiatric disorders (24). In the genetic laboratory routine, the identification of VUS at such genes could significantly answer some clinical phenotypes when other health conditions are part of the patient’s clinical phenotype.

To understand genetic differences and similarities found in ASD subcategories combined at DSM-5 (*The Diagnostic and Statistical Manual of Mental Disorders*), patients who have been previously diagnosed with autistic disorder, pervasive developmental disorder not otherwise specified (PDD-NOS), and Asperger’s syndrome were genetically analyzed for *de novo* mutations (DNMs). Even when only considering the most damaging sort of genetic variants, the results demonstrated that DSM-4 ASD subcategories share many more similarities than differences, considering genes, gene expression, and molecular pathways (25). Similar data, regarding the genetic differences observed between ASD subcategories from inherited genetic variants, should be considered to train predictive tolls concerning clinical features, severity, and outcome in ASD as more genotyping–phenotyping data is being produced.

## Tools for Unveiling the Genetic of Autism Spectrum Disorder

As most clinical cases are associated with a broad spectrum of developmental delay and autistic features, it is also critical to apply a comprehensive genetic analysis approach to increase performance and diagnostic yield. One of the main reasons for the unfeasibility of the genetic panels for ASD is because of the variety of genetic profiles and phenotype diversity in the spectrum, contributing to the popularization of WES and WGS (26). WGS is an ideal option to analyze ASD genotypical variety and complexity, not only due to the possibility of investigating the important regulatory intergenic region (27, 28), but also because of improved sequence coverage comparing to WES low coverage regions affecting essential ASD-related genes (16, 29, 30). Besides, WGS becomes more elucidative if realized in parallel with WGS of the proband’s parents. Despite some criticism regarding cost-effectiveness of the utilization of NGS as an auxiliary tool to support the diagnostic of ASD and neuropsychiatric conditions, recent investigations have demonstrated that even when there are negative or unknown results associated with WES, patients’ clinical trajectories are significantly better, further treatment and healthcare costs are reduced even after unclear genetic investigation (31–35). As the NGS technology costs are significantly decreasing and genetic databases are improving as more patients are sequenced, the cost–benefits relationship is tending to become even better (36).

The genetic variants found are matched with disease-causing mutations from specific databases and are also analyzed using a set of distinct computational tools to predict potentially deleterious DNMs. Although the selection of optimal methods could accelerate the identification of deleterious variants, the absence of a gold-standard approach makes the task of investigating these variations a challenging process. Considering that, some methods rely on the analysis of the effects caused by the mutation in the protein structure, such as SNPMuSiC (37). The approach is a stability-driven knowledge-based classifier that uses protein structure, artificial neural network and solvent accessibility-dependent combinations of statistical potentials to predict whether destabilizing or stabilizing mutations are disease-causing.

Another approach, PolyPhen/PolyPhen-2 (Polymorphism Phenotyping v2), predicts the possible impact of amino acid substitutions on the stability and function of human proteins using structural and comparative evolutionary considerations by annotating the mutation using distinct databases. The tool combined all information and used a prediction method that employs machine-learning classification to estimate the probability of the missense mutation being damaging (38).

Other similar approaches were also developed to annotate and analyze variants, such as SnpEff, which predicts and classifies genetic variants accordingly, i.e., to a synonymous or non-synonymous amino acid replacement, start codon gains or losses, stop codon gains or losses, or frameshifts (39). Another tool, MutationTaster, investigates and scores functional consequences of amino acid substitutions, short insertion or deletion (indel) mutations, variants spanning intron–exon borders, intronic, and synonymous alterations (40, 41).

Another tool, SIFT (Sorting Intolerant From Tolerant), predicts whether an amino acid substitution affects protein function. The tool can distinguish between functionally neutral and deleterious amino acid changes, which was previously validated on mutagenesis studies and human polymorphisms (42).

Other computational tools like SIFT and PolyPhen are based on a combination of methods and are used in prioritizing changes that are likely to cause a loss of protein function. However, their low specificity requires further evidence to support or refute pathogenicity that should be sought before reporting novel missense changes (43).

Recently, 23 distinct computational methods were analyzed to evaluate the performance measures using independent benchmark datasets correlated with disease-causing genetic variants (44). The analysis demonstrated that some methods showed different performances under different conditions, and the specificities were lower than the sensitivities for most of them. It was found that the combination of the software REVEL (45) and VEST3 (46) (i.e., ReVe) showed the best overall performances with all others being the benchmark data, including DNMs.

Although several computational tools have been widely used to predict potentially deleterious DNMs, and the previous comparative analysis suggests that more rigorous analysis is necessary to distinguish pathogenic variants, and consequently can accelerate the better identification of deleterious variants.

## Genetic Databases for Autism Spectrum Disorder

The elucidation of the genetics behind ASD has been contributing significantly to phenotype elucidation. The last decade produced a precious amount of valuable information, which had to be systematized in ASD genetic research and public databases. There are three public databases specialized in genetic variants associated with ASD. SFARI is currently the main source for curated data categorizing associated risk genes with different scores and storing several genetic variants described in the scientific literature (1). AutDb (47) and AutismKB (48) also represent relevant data collected to help genetic curation in ASD. Each database uses different categories and rates evidence in diverse strategies to interpret the relevance of several genes. Considering all databases and recently published articles, there are close to a thousand genes already associated with ASD and related neuropsychiatric conditions.

Several other databases are used to support the classification and clinical impact of genetic variations found within an individual with ASD. The Human Gene Mutation Database (HGMD^®^) is composed of a set of germline mutations correlated with human inherited diseases (49). Another interest databank is denovo-db (http://denovo-db.gs.washington.edu), which provides standardization of annotation and improves accessibility for independent studies reported by the literature. This bank comprised more the 23 thousands of trios, and detailed information about variant information (chromosome location, change, type); detailed annotation at the transcript and protein levels; severity scores; frequency; validation status; and, most importantly, the phenotype of the individual with the variant (16). Another relevant resource is the databank, NPdenovo, composed by DNMs obtained from many studies on neuropsychiatric disorders, that have utilized massive trio-based WES and WGS (50). NPdenovo has 17,104 DNMs from 3,555 trios across four neuropsychiatric disorders: ASD, epileptic encephalopathy, intellectual disability, and schizophrenia, in addition to unaffected siblings (control) from 36 studies by WES/WGS. Another recent database for ASD research is the National Database for Autism Research (NDAR) that contains an extensive collection of clinical and behavioral assessments and health outcomes from novel interventions. NDAR has a global, unique patient identifier that can be linked to aggregated individual-level data, including genetic data, for hypothesis generation and testing, and for replicating research findings (51).

From a different perspective, dbMDEGA databank was created based on a meta-analysis investigation of brain gene expression profiles from reported human ASD expression datasets and knock-out mouse ASD model expression datasets. The databank allows accessing of the differentially expressed genes in the brain of individuals with ASD (52).

Other databanks in ASD have also showed their value when used in combination to help interpret specific mutations of individuals with ASD, such as the interactive autism network (IAN) (53), the Autism Treatment Network (ATN) (54), and PEDSnet (55). They have data from large cohorts of children with ASD but store different information. IAN has dedicated to saving patient-reported measures/phenotyping, ATN has data from clinical characterization, and PEDSnet has data from health care encounters and electronic medical record data. Although presenting strengths and weaknesses, these three databases can be better exploited when used together to interpret a genetic mutation in an individual with ASD (56).

## Autism Spectrum Disorder Genetic Interpretation and Curation

Based on databases generated from several research groups, it is possible to incorporate gene expression data from brain transcriptomes of psychiatric patients to gene pathways associated with ASD, in order to produce more robust evidences and valuable information, to curate and interpret respective VUS in routine NGS analysis (9, 57–59).

Other critical features to analyze during gene curation and interpretation in ASD and neuropsychiatric conditions are genetic constraints, haploinsufficiency, evolutionary conservation and population frequency of genetic variants. Considering population frequency, when genetic variants are extremely rare or even never reported at current databases, such as gnomAD (The Genome Aggregation Database) (60), this is an important indication that might suggest pathogenicity in neurodevelopmental and psychiatric disorders, since low reproductive fitness is highly associated with these disorders. Different research groups demonstrated genetic intolerance to loss of function, such as RVIS (Residual Variation Intolerance Score) and pLI (Probability of Loss-of-function Intolerance), and sequence constraint are significantly associated with ASD and neuropsychiatric conditions like intellectual disability, ADHD, schizophrenia, and bipolar disorder (61–63). Not only genetic variants, related to loss of function intolerance, are significant in constrained genes, but also extremely rare variants never reported before in any public database, which might suggest a similar effect as loss of function alterations. Additionally, a significant association between constrained and loss of function intolerant genes with more severe comorbidities was already demonstrated, being most of these genes described previously as ASD risk genes and sensible to DNM (26).

It is also important to mention that more severe clinical conditions and comorbidities tend to carry a higher genetic burden with several additional hits, including CNVs or rare inherited genetic variants, which are most of the time identified as VUS in neurotypical individuals, but in a significantly lower number (64). Another consistent strategy about genetic constraint is the identification of a specific constrained region in coding genome, not only in loss of function intolerant genes but conserved coding regions and protein domains critical to protein structure and function (65). All these strategies considering genetic constraint data could be combined to elucidate and distinguish VUS, with clinical relevance for ASD and related neuropsychiatric conditions during the genetic curation process.

Last, it is crucial to consider that even sophisticated strategies used to reclassify VUS are not enough for some inconclusive cases, due to multiple and complex genetic profiles recently discovered. Genetic alterations at noncoding regions correspond to a fraction of ASD patients with inconclusive test results. Recent researches identified the relevance of noncoding DNMs at promoter regions (27) and inherited cis-regulatory variants (28), affecting ASD risk genes, which are loss of function intolerant (pLI > 0.9). Unfortunately, the identification of such variants is still far from clinical routine, since it requires WGS with high genome coverage and sophisticated software analysis. Other challenge for clinical routine and relevant genetic events associated with ASD are postzygotic mosaic DNMs, which represent 5% to 20% of total DNMs according to recent data (66–68).

## Conclusions

Considering all these pieces of evidence, they corroborate with the idea that genetic variants associated with neurodevelopmental and psychiatric conditions have a significant impact on reproductive fitness, not only due to rarity and constraint, but also when multiple hits are observed (7). ASD is a complex ND counting with 70% to 90% of heritability rating. In the genetic interpretation process for Mendelian conditions, keeping a focus on the combination of phenotyping and genetic curation could improve diagnostics and provide more relevant results, which will be returned to clinic practitioners (69). A large number of ASD affected individuals tend to have not only extremely rare variants in ASD associated genes, but additionally, some patients with severe phenotypes could have multiple hits accumulating and additively contributing to genetic burden (63, 64). Future investigations and development of novel approaches to identify and calculate such genetic burden, perhaps something similar to the polygenic risk score, but considering rare and ultra-rare variants, would be a breakthrough for the genetics of ASD. For ASD and related neuropsychiatric conditions, the strategy of clinical phenotyping and collaboration during the curation process could provide even more information relevant to genetic complexity and could provide a strong background for patient stratification.

## Author Contributions

We state that all authors have read the paper and approved the data and the conclusions presented therein, and all authors have contributed to the present manuscript with equal effort.

## Conflict of Interest Statement

The authors declare that the research was conducted in the absence of any commercial or financial relationships that could be construed as a potential conflict of interest.
